# Progress in Medicine

**Published:** 1899-06-24

**Authors:** 


### Progress in Medicine.

### DISEASES OF THE RESPIRATORY SYSTEM.

(*Continued from page* 198.)

**Tuberculin.**—Aitkin[17] concludes that tuberculin is of inestimable value in diagnosing tuberculosis in early stages; it is of equal value in discriminating between this affection and others which closely simulate it. In some cases of beginning tuberculosis it is a remedy which possesses curative powers. In tuberculous glands and in local skin tuberculosis the diseased condition is usually at once relieved. Its greatest value at the present time is its use as a diagnostic means.

**Thiocol in Phthisis.**—Soon after the introduction of creosote in the treatment of phthisis, endeavours were made to find substances which, possessing the same therapeutic value, did not show the bye-effects which sometimes prevent the use of this remedy. Carbonate of creasote and guiaicol have been extensively employed. Thiocol, the most recently introduced substitute, has some obvious advantages. Chemically, it is the potassium salt of guiaicol sulphonic acid, is a white powder, not affected by air, easily soluble in water, and does not act as a corrosive on animal mucous membranes even if used in very concentrated solutions. This ready solubility in water is a very great advantage. Rossbach[18] has recently ascertained by experiments on animals that it is readily absorbed by the animal body, and is non-toxic when administered either by the mouth or subcutaneously. As much as 450 grammes were given daily to a dog without ill-effects.

Numerous cases of phthisis have been treated by the remedy, in daily doses of from 100 to 200 grains, with uniformly good results.

**Polmonary Actinomycosis,** according to Sabrazés and Cabanés,[19] occurs in from 12 to 15 per cent. of cases of actinomycosis in general. The lungs may be invaded primarily or secondarily. The primary lesion may be in the œsophagus, posterior mediastinum, or even the abdomen, more rarely in the trachea or cervical cellular tissue. In other cases a primary broncho-pleural actinomycosis may be present. The process is essentially ulcerative, and three stages are described—broncho-pulmonary, pleural, and fistulous. The actinomycotic lung shows on section large numbers of abscess cavities, and large tracts of fibrous tissue, not unlike caseating tubercle. In the cavity wall small yellow bodies may be seen in great numbers, which are the nodules of actinomyces. The process, though in a few cases rapid, as a rule spreads over years. The onset is insidious. As a rule the apex is unaffected, and the lateral and posterior parts of the lung are the parts usually affected. As the disease progresses the patients are troubled by incessant cough, loss of weight, and severe pain. The sputa are generally extremely fœtid, but sometimes milky white, almost like brain pulp, due to the large amount of fatty cells they contain; occasionally they are streaked with blood, but they do not contain elastic tissue probably from the fact that the sclerotic process is so early and so extensive. The presence of the organism can usually be demonstrated. When the disease reaches the chest wall a boggy red swelling with surrounding œdema appears. Exploration with a needle rarely shows the presence of fluid, but incision reveals glutinous pus containing yellow grains. Severe bone lesions affecting ribs or vertebræ may be caused by the extension of the disease. Internal treatment is unsatisfactory, but good results have been obtained by the administration of iodide of potash and eucalyptus. When the thoracic wall is affected surgical interference is necessary, though generally unsatisfactory. Cleaning out dead tissue and necrotic bone with scraping of the surface of the diseased tissue has led to good results in a few cases.

**Cheyne-Stokes Respiration.**—Whitehead[20] records some fresh cases of ocular phenomena associated with Cheyne-Stokes respiration and gives a summary of our knowledge of the subject. In the majority of cases of this periodic alteration of the rhythm of respiration no eye symptoms whatever have been observed, but in a certain number definite disturbances of the intra and extra-ocular muscles have been seen. These disturbances are of considerable variety, and they may sometimes be observed in what may conveniently be called a Cheyne-Stokes state or condition apart from any disturbance in the rhythm of respiration. Every variety of case may be seen, from a simple Cheyne-Stokes breathing to the complete picture of persistent and regular Cheyne-Stokes respiration, attended during the pause by unconsciousness, closed eyelids, contracted pupils, and lateral conjugate deviation of the eyeballs, the pulse during this period being frequent, small, and of moderately high tension. At the end of some seconds consciousness returns, the eyes open, the pupils slightly dilate, the pulse loses some of its tension, and a superficial respiration follows.

The cause of Cheyne-Stokes breathing is as yet unknown. It is supposed that some special condition of the respiratory and other centres is responsible for the whole series of phenomena. The respiratory centre is automatic, and when freed from the higher centres becomes periodic in its action. In hibernating animals and in man during deep sleep, for instance, periodic breathing may often be seen. If by cerebral pressure, by uræmic blood, by deficient arterial supply, or by the administration of certain drugs the control of the higher centres is lessened or abolished the lower centres tend to show periodic variations in their activity. This is chiefly seen in the action of the respiratory centres, but other centres, such as those of the intra and extra-ocular muscles, may also participate.

[17] Aitkin, Denver Med. Times, No. xviii., 1898. [18] Rossbach, Therapist, Mar. 15, 1899. [19] Sebrazés and Cabanés, Rev. de Méd., Jan. 10, 1899. [20] Whitehead, Lancet, Feb. 25, 1899.

### DISEASES OF CHILDREN.

**Tuberculosis and Milk.**—In the course of the recent tuberculosis crusade it has been alleged on very high authority that tabes mesenterica and tuberculous meningitis are increasing in infants as the direct result of an infected milk supply. This theory explains the origin of tuberculosis in infants by the assumption that the tubercle bacilli are conveyed to the intestines by milk from tuberculous cows. As the result of pathological investigation some doubt has been thrown on this plausible theory by various writers. Dr. Walter Carr[1] shows from the post-mortem records at the Victoria Hospital for Children that tabes mesenterica is rare in children under two years of age, and almost unknown in those under one year of age. He holds that the primary source of infection is much more frequently through the respiratory than through the alimentary tract. From an examination of the post-mortem records at Paddington Green Children's Hospital, Dr. Leonard Guthrie[2] shows that thoracic tuberculosis was most prominent and probably primary in 54·5 per cent. of the cases, and abdominal phthisis in 24·6 per cent. He points out that, in addition to direct entry of the bacilli into the lungs through the air passages, they may reach the thoracic glands through the pharynx, tonsils, and œsophagus above, and through the lymphatics of the intestines and the abdominal glands below. Confirmation of these views comes from America. Dr. Bovaird[3] concludes from his experience in the autopsy room of the New York Foundling Hospital that the primary lesion of tuberculosis in children is regularly found in the bronchial lymph glands or in the lungs. Combining his statistics with those of Northrup on the same subject, he shows that, out of a total of 220 autopsies, in 148 cases infection was by the respiratory tract, in 3 cases infection was by the mesenteric lymph glands, and in 34 cases infection was not determined. He further points out that the early manifestations of tuberculosis in children are extremely indefinite and uncertain, and tuberculosis of the bronchial glands cannot be diagnosed with certainty. He agrees with Dr. Guthrie in thinking that this latent bronchial tuberculosis is frequently aroused and disseminated by the invasion of another disease, such as measles. In view of these pathological facts, too much stress must not be laid on the death returns of the Registrar-General as proving that tabes mesenterica from tuberculous milk is a common and fatal malady in infancy.

**Pericarditis.**—Out of 66 cases of pericarditis in childhood which have been under the care of Professor Baginsky,[4] no less than 20 occurred during the first year of life. Serous pericarditis is usually associated with rheumatic polyarthritis, and is accompanied by dyspnœa, pyrexia, and rapid cardiac action. The patient ought to be kept recumbent in bed, and icebags applied continuously over the heart. Purulent pericarditis is a secondary affection, usually very difficult to diagnose, and extremely fatal. An attack of rheumatic pericarditis leaves permanent changes, enlargement of the heart, a heaving impulse, an apical systolic murmur, and an accentuated second pulmonary sound. Recurrent attacks are common, and evidences of progressive deterioration are present, namely, pallor, dyspnœa, cough, bulging of the ribs, and stormy cardiac action. The malignancy of this condition is due to the association of endocarditis and myocarditis with the pericarditis. Salicylates are useless in the treatment of rheumatic pericarditis, but the author finds much benefit in the subsequent cardiac failure from a combination of full doses of diuretin with strophanthus or digitalis.

**Thread Worms.**—Out of 200 consecutive necropsies on children, Dr. G. F. Still[5] found thread worms in the intestine in 38 cases, and in no fewer than 25 of these the worm was present in the vermiform appendix. Sometimes the appendix was the only part in which they were found, and from their immature character and the large numbers present he is inclined to believe that the appendix may be a breeding-place for the oxyuris vermicularis. He combats the view usually held that each thread worm in the intestine has been swallowed as an ovum, and points out that there is no evidence of any source of supply which would result in the scores of thread worms frequently passed. The worms in the appendix may be a source of irritation, producing catarrh and the symptoms of appendicitis. The dislodgement of the worms from the appendix will naturally be a matter of some difficulty, and the author believes that santonin by the mouth will be found more valuable than salt-and-water injections into the bowel.

**Gonococcus Joint Disease.**—Attention has been called to this affection occurring in infants as the result of purulent ophthalmia by Mr. Clement Lucas.[6] This was a second communication, and was the result of a study of 23 collected cases. The joint disease presented itself in two forms: (1) As a very acute arthritis accompanied with much swelling, tenderness, and redness; and (2) as a subacute synovitis, with effusion and pain on movement, but little or no redness. The left knee-joint was the part most frequently affected. Complete resolution might be anticipated, and the inflammation usually ran a course of from three to five weeks.

**Progressive Ossifying Myositis.**—This condition is found by Missim[7] to develop usually in childhood, although it may appear in adult life. It is characterised by the deposition of osseous material in the connective tissue of the muscles, in the tendons, aponeuroses, and ligaments. The onset may be acute, with fever, pain, and œdematous swelling. In other cases it is chronic and insidious, one or more swellings being accidentally discovered. Amongst the preceding circumstances regarded as causal are rheumatism and traumatism, but frequently no cause can be discovered. The muscles of the back and neck are the most frequently and the most early affected, and the resulting conditions are ankylosis of the shoulder joint and immobility of the scapulæ and vertebral column. The appearance of the sufferer in a well-marked case is striking, as he is converted into an immovable block. The mental condition and the internal organs are unaffected, the progress of the disease is slow, and death is commonly due to some intercurrent affection, particularly tuberculosis. A well-marked example of this affection is recorded by Crawfurd and Lockwood,[8] in a paper fully illustrated with photographs and skiagrams. The onset of the illness in this patient dated from a fall on the shoulder at the age of two and a half years. There were present some congenital deformities in the feet, namely, an absence of the ungual phalanges of the third and fourth toes of the left foot, and a projection outwards of the great toe under the second toe on each foot. This condition has frequently been observed, and the authors find an explanation of it in a synostosis of the metatarsal bone with the first phalanx, as shown in a skiagram.

**Nephritis Dependent on Gastro-Enteritis.**—The occurrence of a form of pneumonia secondary to septic processes in the intestinal canal of infants has been fully recognised; but, according to Koplik,[9] nephritis may result from a similar condition. The clinical symptoms of nephritis may be slight or severe. The patient is both restless and drowsy; that is to say, periods of the one condition alternate with periods of the other. Constant vomiting, unaffected by the usual treatment of gastro-enteric trouble, is another prominent feature, and is to be regarded as a manifestation of uræmia. A varying amount of œdema, showing itself on the inside of the thigh, or the front of the leg, or the dorsum of the foot, may be present. The urine in marked cases is extremely scanty, loaded with urates; and, although only a mere trace of albumen is present, there may be casts of all kinds in abundance with blood cells and renal epithelium. The author recommends in such cases a diet of albumen water or diluted beef juice, gastric lavage and rectal irrigation, and bismuth in large doses. He especially deprecates the use in such cases of opium, calomel, and corrosive sublimate, as they are positively injurious.

**Cirrhosis of the Liver.**—An interesting example of this affection in a child of six years is recorded by Hunter and Workman.[10] The patient lived only two and a half hours after admission to the hospital, and from a hasty examination tuberculous peritonitis with infiltration of the lungs was diagnosed. The illness had existed for two months, the first changes noted being swelling of the abdomen and yellowness of the skin, and later there had been constant diarrhœa. At the autopsy the liver was found to be markedly irregular in contour and nodulated, which condition was due to pronounced cirrhosis mostly of the multilobular type. Examination of the parents elicited the fact that the child had consumed considerable quantities of alcohol, commencing with teaspoonful doses of whisky when as a baby he was suffering from bronchitis, and later getting a "little drop" whenever the parents were refreshing themselves. The authors very guardedly do not assert that alcohol was the direct cause of the cirrhosis, but they see a causal relationship between the two.

**Inguinal Hernia in Childhood.**—At the Medical Society of Victoria a discussion has taken place on the question as to whether all congenital hernias should be operated on or not. Mr. F. D. Bird[11] said that surgeons generally believe in the wearing of a truss in early childhood, reserving operation for exceptional cases. He said that they were justified in this procedure, considering (1) the very strong inherent tendency of the funicular process to become obliterated; (2) our ignorance on the subject of the period of its ordinary closure; and (3) the variation in the time of the descent of the testicle. He could not overlook the facts that in a small child the operation was not of the easiest, and that the separation of the sac was a delicate procedure, very apt to cause injury to the cord or testis.

[1] Lancet, Dec. 17, 1898. [2] Ibid., Feb. 4, 1899. [3] Med. Rec., Mar. 18, 1899. [4] Berlin Klin. Woch., Nos. 48 and 49; and Treatment, Jan. 12, 1899. [5] Brit. Med. Jour., 1899, I., p. 898. [6] Lancet, Jan. 28, 1899. [7] Jour. de Med., 1898; and Epit. Brit. Med. Jour., April 1, 1899. [8] Lancet, 1899, I., p. 1,021. [9] Med. Rec., 1899, I., p. 451. [10] Glasgow Med. Jour., Feb., 1899. [11] Inter. Med. Jour., Austral., Sept. 20, 1898.

